# The legal and economic aspects of the “Esports Illusion”—why competitive gaming fails to become an independent industry

**DOI:** 10.3389/fspor.2025.1636823

**Published:** 2025-10-03

**Authors:** Pál Novák, Balázs Hohmann, Dávid Sipos, Gergely Szőke

**Affiliations:** ^1^ Faculty of Health Sciences, University of Pécs, Pécs, Hungary; ^2^ Department of Technology Law and Energy Law, Faculty of Law, University of Pécs, Pécs, Hungary; ^3^ Department of Medical Imaging, Faculty of Health Sciences, University of Pécs, Pécs, Hungary

**Keywords:** eSports, intellectual property, legal asymmetry, digital platforms, economic consolidation, publisher governance

## Abstract

**Introduction:**

The esports industry has experienced rapid growth over the past decade; however, recent trends indicate a phase of consolidation rather than continued exponential expansion, often referred to as the “Esports Winter.” This paper examines the hypothesis that the lack of a coherent legal framework, combined with the industry's economic model and game publishers' dominance over esports competition rights, significantly influences the market correction and restructuring of the esports ecosystem.

**Methods:**

This study employs a comparative legal analysis, focusing on intellectual property rights, contract law, and the regulatory asymmetries that define the esports ecosystem. Additionally, economic data from market reports and financial disclosures are used to examine the financial structures controlled by game publishers and to contextualize the industry's consolidation within the broader video game market.

**Results:**

Findings reveal that the key factor in esports consolidation is the legal and economic dominance of game publishers, who retain full control over competition rights and financial structures. Unlike traditional sports, where governing bodies regulate commercial rights, esports remains publisher-driven, making independent growth and financial stability contingent upon their strategic priorities. While some esports ecosystems, such as those surrounding Counter-Strike 2 and Dota 2, allow for third-party tournament organization, the industry as a whole remains structurally dependent on game publishers. These companies, despite having the exclusive legal rights to esports competitions, are not primarily motivated to develop the industry beyond its function as a marketing and engagement tool for their games. Consequently, while publishers do not actively restrict independent esports growth, the lack of alternative legal frameworks prevents other stakeholders from establishing a broader, more autonomous regulatory structure. Moreover, esports revenues account for less than 1% of the global video game industry's total income, indicating that esports is a supplementary rather than a standalone sector.

**Discussion:**

The study concludes that esports' current challenges stem from its integration within the video game industry rather than being an independent sports-like entity. The ongoing consolidation phase reflects a market correction rather than an existential crisis. Unlike traditional sports, esports lacks an overarching regulatory authority, as all competition rights remain within the control of game publishers. This structural characteristic is unlikely to change, given that intellectual property rights grant publishers exclusive authority over how their games are used in competitive settings.

**Conclusion:**

Rather than representing an industry-wide crisis, the current phase of esports reflects a long-term stabilization process in which it remains an extension of the broader video game market. While legal frameworks could theoretically alter the regulatory landscape, any fundamental shift would require game publishers to relinquish control over their intellectual property—an outcome that remains improbable. As a result, esports will likely continue to operate within publisher-driven economic models rather than evolving into an independent, traditionally governed sports industry.

## 1 Introduction

Over the past decade, the esports industry has experienced exponential growth, with competitive gaming emerging as a mainstream form of entertainment and a legitimate professional career path (1, 2). As major publishers and sponsors increasingly invested in tournaments and leagues, many anticipated that esports would develop into a globally recognized, self-sustaining industry, comparable to traditional sports (3). However, recent trends suggest not continued expansion, but rather a phase of consolidation—referred to by many commentators as the “Esports Winter” (4).

While some observers interpret this deceleration as a sign of market instability, this paper argues that the industry is undergoing a structural correction process, adapting to its legal and economic particularities. Contrary to the general narrative prommoted in the media, esports has never been an autonomous sector—and it has not evolved into one. This article aims to examine the structural and legal-economic reasons for that reality.

Unlike traditional sports, where competitions are governed by independent federations separate from commercial actors, the entire structure of esports remains under the control of game publishers. The reason is straightforward: all esports events are conducted through video games—proprietary software products—which are legally protected as intellectual property (5). This structural dependency raises fundamental questions about whether esports can ever function as a standalone industry or whether it will remain a permanent extension of the video game market.

The primary aim of this study is to examine how the legal and economic control exercised by publishers shapes the ongoing consolidation of the esports industry. Through the analysis of intellectual property regulations, licensing frameworks, and economic structures (5, 6), the study investigates whether current trends represent a temporary downturn or the onset of a longer-term stabilization. It further explores whether regulatory intervention or alternative governance models might realistically challenge the current publisher-centric paradigm.

Research Questions:

1. To what extent does the intellectual property held by publishers shape the structure of the esports industry?
2. How does the legal and economic role of publishers differentiate esports from traditional sports in terms of copyright and regulation?
3. Should the current consolidation phase be interpreted as a sign of decline, or rather as a market correction stabilizing esports as part of the broaeder video game ecosystem?

By addressing these questions, this paper seeks to contribute to the ongoing scholarly discourse surrounding esports governance and long-term sustainability.

## 2 Materials and methods

This study employs a qualitative and comparative legal research methodology, complemented by economic data analysis. The primary aim is to explore how legal and economic structures—particularly the dominance of game publishers—shape and are related to the consolidation of the esports industry. The analysis is built around three pillars: intellectual property rights, contractual relationships, and economic indicators relevant to the operation of esports.

### 2.1 Legal analysis

A doctrinal legal approach was used to examine the structural consequences of publisher control over the competitive framework of esports. This included a comparative review of End User License Agreements (EULAs), terms of service, and tournament licensing policies of major publishers such as Riot Games, Valve, and Activision Blizzard, as these companies publish some of the most popular and commercially influential esports titles globally, including League of Legends, Valorant, Counter-Strike 2, Dota 2, and Call of Duty (7, 8). Special attention was paid to how these contractual frameworks influence the organization of third-party tournaments, player rights, and league governance (6, 9).

The study also considered specific examples of national regulation, with particular attention to France's esports legislation (10), which legally recognizes professional video game competitions and establishes employment standards for professional players. These examples were used to assess the extent to which national legal systems are capable of addressing the imbalances present in the global esports ecosystem (11).

### 2.2 Economic contextualization

To better understand the financial dynamics of the industry, the research analyzed secondary data from market intelligence reports (e.g., Newzoo Global Esports Market Report), publicly available financial statements, and independent industry analyses. Key indicators—including total esports revenues, the proportion of revenue from sponsorship and media rights, and the share of esports within the global video game market—were used to contextualize the legal findings (12–14).

This economic perspective allowed for a comparative analysis between the structural characteristics of esports and traditional sports business models, with particular focus on regulatory independence and revenue distribution.

### 2.3 Scope and limitations

This study does not rely on empirical interviews or primary data collection. Instead, it draws on legal statutes, licensing documents, and secondary financial data published by industry analyts and observers. This approach ensures a focused analysis of the legal-economic structures of esports; however, it does not capture the subjective experiences of players, teams, or tournament organizers. Furthermore, given the rapidly evolving nature of the esports industry, the study reflects a snapshot of regulatory and economic conditions as of 2024–2025. While certain variables may shift over time, the core legal and structural dynamics identified—particularly the intellectual property-based control of publishers—are deeply embedded in international frameworks and are unlikely to change in the foreseeable future. Therefore, despite the methodological limitations, the findings offer a robust and well-grounded account of the current structural realities of the esports ecosystem.

## 3 Results

This section presents the findings of the legal and economic analysis conducted to examine the structural dynamics of the esports industry. The results are organized thematically in accordance with the dual focus of the study: legal frameworks and economic structures.

### 3.1 Analysis of the legal primacy of publishers

One of the most fundamental legal characteristics of the esports ecosystem—often overlooked in surface-level analyses—is the structural exclusivity created by international copyright law. Unlike traditional sports, where the rules of the game and the right to organize competitions are not owned by any individual entity, video games are protected as copyrighted works. This includes the underlying software code, visual assets, audio, gameplay mechanics, and branding elements. As a result, any public performance, broadcast, or reproduction of the game requires explicit authorization from the copyright holder, which in the case of esports is almost always the game publisher (5, 6).

This legal protection is not a domestic anomaly but a universal principole rooted in international treaties. The Berne Convention for the Protection of Literary and Artistic Works (1886) mandates automatic copyright protection in all member states (included 181 member countries worldwide, including all EU member states but also the United States, Canada, China, Japan, Brazil, Australia, South Korea etc.), and it recognizes the exclusive rights of authors over reproduction, adaptation, and public performance of their works (15). The TRIPS Agreement (1994), adopted under the auspices of the World Trade Organization, binds its signatories to enforce the standards of the Berne Convention and provides mechanisms for international dispute resolution (16). Furthermore, the WIPO Copyright Treaty (1996) expands these protections into the digital realm, confirming that computer programs and interactive works are fully protected literary creations, and establishing the exclusive right to make them available to the public via digital networks—including competitive broadcasts and streaming (17).

In parallel, the relationship between the user (player) and the copyright holder is contractually defined through End User License Agreements (EULAs). These agreements grant users strictly limited, personal-use licenses to interact with the game software under predefined terms. Importantly, EULAs typically prohibit any form of public competition, commercial exploitation, or broadcasting without prior written consent from the publisher (7, 8). Thus, while players may possess a license to use the game, they have no legal right to independently organize or broadcast a tournament involving that title.

This legal structure creates a dual-layered framework of control: first, the objective legal protection offered by international copyright regimes; second, the subjective contractual restrictions imposed through EULAs. Together, these ensure that game publishers retain not only creative ownership, but exclusive procedural authority over how, when, and by whom their games are used in a competitive context. This stands in stark contrast to traditional sports, where no single entity owns the concept or rules of a game like football or athletics, allowing for pluralistic organization and autonomous governance.

In essence, it is this intellectual property framework that legally prevents esports from evolving into a traditional sports-like system governed by independent federations or associations. The current publisher-centric structure is not simply a commercial preference—it is a legally entrenched status quo, backed by globally binding treaties and enforceable through both civil and international legal mechanisms. Any structural transformation of the esports ecosystem would therefore require either a voluntary relinquishing of rights by publishers (which is unlikely), or a radical shift in international copyright doctrine (which is unprecedented). As such, the consolidation of esports must be interpreted as a stabilization within the bounds of an entrenched legal monopoly, not as a failure to emulate the sports world.

### 3.2 Publisher governance and legal asymmetry in esports regulation

The analysis confirms that all major esports titles—including League of Legends, Counter-Strike 2, Dota 2, Valorant, and Call of Duty—are fully controlled by their respective publishers, who retain all intellectual property rights. This includes exclusive authority over tournament organization, broadcasting rights, and licensing conditions. These rights are contractually established through End User License Agreements (EULAs) and tournament-specific terms of service. For instance, Riot Games explicitly prohibits the use of League of Legends content for tournament organization without prior written consent (18). Valve applies a more flexible approach, allowing community-run tournaments for Dota 2 and Counter-Strike, but still reserves final control via its Steam User Agreement (19).

This publisher-centric model stands in stark contrast to traditional sports, where national or international federations—such as FIFA (20, 21), FIBA (22), or the IOC (23)—act as independent regulatory bodies. In esports, there is no neutral third party capable of standardizing competition formats, player rights, or dispute resolution mechanisms. As a result, tournament organizers, teams, and players operate under contractual asymmetry, where publishers unilaterally define all operational and legal conditions without any obligation to consult stakeholders.

In traditional sports, international competition frameworks—such as the Olympic movement, FIFA tournaments, or World Athletics events—legitimize the titles awarded through globally recognized mechanisms. To become the world's top pole vaulter, for example, one must not only achieve elite physical performance but also obtain a competition license, participate as a member of a national federation, and comply with conditions like doping controls, medical eligibility, and qualification standards. These criteria are not enforced through legal coercion but derive from the symbolic authority of global sport institutions, whose championship titles are widely accepted as markers of supremacy.

By contrast, no such legitimizing mechanism exists in the world of esports. To become a “champion” in a given game, one must win a tournament either organized or approved by the game's publisher. Thus, publishers hold not only legal but also symbolic monopoly over competitive legitimacy. There is no independent third party whose normative framework enjoys universal recognition. Accordingly, no superior regulatory authority exists that could limit publishers' jurisdiction over their respective titles.

### 3.3 National legislation: fragmented and limited scope of intervention

Although some countries have made efforts to define and regulate esports, these initiatives remain fragmented and are largely ineffective in constraining the dominance of game publishers. The most notable example is France, where regulators recognized the issues outlined above—particularly the fact that the internationally recognized copyright status quo makes it unrealistic to limit publishers’ rights. Instead, the French approach focuses on the primary setting in which esports takes place. The 2016 law (Loi n° 2016-1321) legally recognizes professional video game competitions, defines their legal status, and regulates the employment relationships of professional players (10). However, this regulation does not alter the underlying rights structure: publishers continue to hold exclusive rights over game content, tournament organization, and monetization. The law merely regulates the practical realities surrounding competitions that have already been authorized by the publishers, defining the rights and obligations of players and the organizations employing them. Thus, although the regulation creatively protects players, it does not change the foundational asymmetry.

In South Korea—where esports has become a prominent national industry—multiple public-private partnerships and talent development programs are in place. Nonetheless, there is still no legislation that limits the control of publishers over esports competitions. Therefore, even in jurisdictions where national-level regulation exists, no autonomous governance model has emerged that resembles the institutional independence seen in traditional sports (11).

### 3.4 Revenue structure of the esports industry

According to Newzoo's 2024 Global Games Market Report, the global video game industry is projected to generate approximately $187.7 billion in revenue in 2024, representing a 2.1% year-on-year growth. This figure underscores the substantial scale of the video game market (20).

In contrast, the esports sector, while experiencing growth, remains a relatively small segment of the overall industry. Estimates suggest that global esports revenues were around $1.64 billion in 2023, with projections indicating continued growth in the coming years. This comparison highlights that esports accounts for less than 1% of the total video game industry revenue, reinforcing the view that esports serves more as a complementary component rather than a primary revenue source for publishers (14).

The revenue structure of esports is heavily reliant on sponsorships and media broadcasting rights. For instance, in 2021, sponsorships accounted for approximately 62% of global esports revenues, while media rights contributed about 19%. Publisher revenues directly related to esports—such as merchandise, ticket sales, or in-game monetization linked to tournaments—remain minimal compared to the income generated from game sales and microtransactions (12).

These figures support the assertion that, despite its high visibility, esports functions primarily as a marketing strategy for publishers, aiming to enhance user engagement and extend the lifecycle of their games, rather than serving as a standalone profit center.

### 3.5 Consequences of publisher-driven economic models

Since direct revenues from esports remain relatively low and publishers retain full control, there is little financial incentive for them to develop autonomous ecosystems or transfer authority to third parties. Instead, esports events often serve as tools for brand building and community engagement, helping sustain interest in the game and support long-term monetization (4).

Or instance, the League of Legends World Championship, organized by Riot Games, is not primarily designed to be profitable, but functions as a cornerstone of the company's global brand strategy (14). Similarly, Valve's tournament, The International, for Dota 2, is known for its exceptional community engagement driven by in-game crowdfunding (24). While the company maintains significant authority over the event's organization and revenue distribution, it also leverages its platform to retain control over broadcasting (14). These examples illustrate that publishers do not treat esports as a profit-driven industry, but rather as a tool for reinforcing the broader game ecosystem.

This perspective further supports the core argument of the present study: esports is not an independent industry but an integrated extension of the video game market.

### 3.6 Absence of independent stakeholder representation

In contrast to traditional sports, where players' unions, independent leagues, and federations ensure a system of checks and balances, esports lacks institutional representation for stakeholders independent of publishers. While there are regional initiatives aimed at protecting player rights—such as the Esports Integrity Commission or certain national player associations—these entities possess no binding legal authority and operate entirely outside the regulatory frameworks defined by publishers (9, 25). The broader literature also confirms that esports lacks consistent disciplinary frameworks, anti-doping policies, and standardized codes of conduct, which further weakens stakeholder protections compared to traditional sports (26). In the absence of legal recognition or enforceable rights, teams and players remain contractually subordinate to publishers. For players, this subordination is twofold: first, they are bound by the End User License Agreements (EULAs) they accept when engaging with the game, which in the case of Blizzard, Valve, or Riot Games titles can terminate a player's career even without explicit cause (5, 7, 8, 27). Second, a separate legal relationship exists between players and the esports organizations that employ them. As a result, players are legally vulnerable both in their contractual relations with publishers and with their teams, without the protection of any independent governing body.

## 4 Discussion

The results of this research indicate that although the esports industry enjoys increasing visibility and professionalization, its structural characteristics fundamentally differ from those of traditional sports. Instead of evolving toward independent governance structures that prioritize player protection and a self-sustaining economic model, esports continues to function as a publisher-driven extension of the video game industry—one that is deeply defined by legal and economic asymmetries.

### 4.1 Legal structure: the limits of intellectual property

The primary legal conclusion is that full control over competition organization stems from the intellectual property rights held by game publishers. This is not a temporary feature of the esports ecosystem, but its structural foundation (1, 5, 6, 17). Unlike traditional sports—such as football or tennis—which are not owned by any single entity and can be freely practiced and organized under independent federations, esports titles are closed, legally protected products. This creates a lasting power imbalance between publishers and other stakeholders, such as teams, players, and tournament organizers (5, 6, 9).

Although numerous efforts have been made to establish neutral regulatory frameworks or player unions, most of these initiatives remain symbolic or advisory in nature, lacking real decision-making authority (28). In the absence of legislative or contractual mechanisms that would compel publishers to share governance, institutional autonomy within esports remains fundamentally limited—or, more accurately, nonexistent.

### 4.2 Economic incentives and strategic priorities

Economic data show that esports plays a supplementary rather than central role in the business models of game publishers. Although esports does generate revenue through sponsorships and media rights, these streams are minimal compared to the revenue from game sales and microtransactions (12, 14). As a result, publishers tend to invest in esports when it serves purposes such as player retention, brand building, or extending the product life cycle—rather than as a standalone source of profit (4, 14).

This functional approach also explains why certain high-profile leagues—such as the Overwatch League (OWL) have been discontinued or restructured. The reason was not a failure of the esports concept itself, but rather that the investment did not meet publishers' expectations. Blizzard Entertainment launched the OWL in 2018 with a franchise-based business model, high entry fees, and a rigid structure. Although it initially envisioned rapid global expansion, the league could not sustain itself in the long term: viewership numbers declined, many live events were canceled due to the COVID-19 pandemic, and the financial viability of the teams became uncertain. In November 2023, Blizzard officially announced the end of the league and introduced a new, more flexible tournament format—the Overwatch Champions Series (OWCS)—to be launched in 2024 (29).

In contrast, ecosystems that allow for community involvement and decentralized tournament organization—such as Valve's approach with Dota 2—have proven to be more sustainable (27). Rather than establishing a franchise model, Valve implemented a point-based qualification system through the Dota Pro Circuit (DPC), which relies on independently organized tournaments. The season culminates in The International, a premier event with a prize pool funded through in-game purchases by the player community (27). This model has proven viable in the long run because it allows for gradual participation, entails lower financial risk, and fosters a more direct connection with the community.

### 4.3 Esports consolidation as structural correction

The current so-called “Esports Winter” is not a collapse but a market correction. Following a decade marked by speculative investments, inflated valuations, and overly optimistic comparisons with traditional sports, the industry is now reverting to its actual structural boundaries. It is becoming increasingly clear that sustained, dynamic economic growth in esports was always an illusion. The realities of publisher control, the absence of institutional autonomy, and limited direct revenue streams all suggest that the “industry”, or rather be called, sector is stabilizing into a position that better reflects its legal and economic foundations (4, 5, 14, 26).

However, this transition does not indicate a loss of relevance. On the contrary, consolidation may contribute to greater sustainability, more realistic expectations, and more stable business models. In previous years, numerous invesitments—especially in franchise-based leagues—were modeled after traditional sports without acknowledging the legal and economic distinctiveness of the esports sector. The case of the Overwatch League exemplifies this misalignment: exorbitantly high franchise fees, a rigid league structure, and a business model built on rapid global expansion failed to yield returns in terms of viewership or sponsorship revenue (29).

Through consolidation, the industry is returning to its roots. Tournament organizers can now focus on reaching their true target audiences—the player communities and fanbases—instead of chasing unrealistic expansion and professional sport status. Publishers, in turn, have the opportunity to optimize their competition structures within the ecosystem of their games, aligning them with community needs rather than investing in unprofitable prestige projects.

Thus, esports should be understood as a long-term, organically evolving medium whose growth and significance depend not on replicating the model of traditional sports, but on adapting to the specific characteristics of the digital economy.

### 4.4 Unrealistic expectations of regulatory reform

Although some experts suggest that regulatory intervention could “liberate” esports from publisher control, such scenarios are largely unrealistic at the international level. Forcing publishers to relinquish their intellectual property rights would not only be legally unprecedented but also economically irrational, as the games themselves constitute the publishers' primary assets (5, 6, 14, 30).

Limited progress may be possible through “soft law” instruments such as ethical codes, best-practice guidelines, or national accreditation schemes [as seen in France (10)]. Some scholars argue that explicitly recognizing esports as a form of sport could provide a more coherent regulatory basis and help address existing legal gaps by aligning it with the frameworks of traditional sports (31). However, these mechanisms can only supplement—not replace—the publisher-centric structure. Even if such instruments were to contain more stringent legal provisions rather than purely ethical recommendations, they would still lack enforceability against copyright holders.

While some commentators envision decentralized technologies as a potential counterweight to publisher control, this notion remains unconvincing in the context of the current legal and institutional landscape. Copyright law itself is rooted in centralized authority: treaties such as the Berne Convention and its revisions (e.g., the 1971 Paris Act) (15) are the product of state-based negotiation and enforcement. Publishers, too, are centralized corporate actors operating under the legal protection of these frameworks. Even communities that may appear decentralized—such as informal player alliances—lack standing to challenge these structures. Most importantly, end-user license agreements (EULAs), which are a prerequisite for launching any esports title, are accepted before any form of community coordination can emerge. Thus, both legal authority and software access are structurally centralized in ways that effectively preclude decentralized alternatives from altering the status quo.

For this reason, the current operational model of esports should not be interpreted as a transitional phase but rather as a durable status quo.

### 4.5 Implications for stakeholders

For teams, players, and tournament organizers, the current structure means adapting to an environment where influence is gained not through institutional autonomy, but through collaboration with publishers (5, 9). Legal literacy, strategic partnerships, and professional management may become more important than formal representation frameworks. For regulators, the challenge lies in promoting transparency and fairness within the boundaries set by publishers' intellectual property rights (5, 6, 10). This lack of structural protections also contributes to broader challenges in career sustainability for esports players, who often face early burnout and short competitive lifespans due to intense demands and limited institutional support (32). The academic and legal discourse must also evolve: esports is not traditional sport, and it cannot be analyzed using models that are not based on software protected by intellectual property law (5, 6). Instead, interdisciplinary approaches are needed—combining intellectual property law, digital platform governance, and economic strategy—in order to meaningfully explore the future trajectory of the industry.

While institutional autonomy remains out of reach for most stakeholders, different levels of collaboration with publishers are emerging as the only viable pathway to operate within the current legal framework. For example, the International Olympic Committee (IOC) has launched the Olympic Esports Games, now scheduled for 2027 in Riyadh (33). The delay from the originally planned 2025 date was reportedly due in part to ongoing uncertainty over which games could be featured—highlighting once again that even global institutions like the IOC must negotiate access with publishers, who retain ultimate control over competitive use of their titles. Similarly, the International Esports Federation (IESF) has attempted to position itself as a global governing body by organizing tournaments and advocating for standardization. However, it continues to lack any real authority over game publishers, and can only operate within the parameters set by them (30). These examples underscore a structural reality: publishers have no economic incentive to invest in partnerships or governance models that do not directly serve their strategic interests.

Although the legal framework grants publishers uniform control, their actual strategies differ substantially in practice. Valve stands out with its model of “controlled openness”: it allows third-party tournament organization only under strict licensing terms, via a Limited Game Tournament License agreement (34). Without meeting these criteria or obtaining explicit permission organizers are not permitted to host tournaments using Valve titles (e.g., CS2, Dota 2) (30) In contrast, Riot Games and Blizzard operate more centralized models, where nearly all competitive activity is directly managed or authorized by the publisher. While Valve thus represents a comparatively flexible outlier, the EULAs and licensing regimes it enforces still reinforce the core legal argument of ultimate publisher control, because at end of the day Valve's permission is still needed by the organizers. It seems it offers more practical flexibility, but all tournament activity still depends on the publisher's discretionary approval.

### 4.6 Summary of interpretations

• Esports is structurally dependent on the intellectual property rights held by game publishers.
• The ongoing consolidation phase reflects a market correction rather than a collapse.
• Publisher incentives prioritize player engagement over direct profitability (This means that for game publishers, esports is not primarily a profit-generating sector in itself, but rather a strategic tool to ensure that players stay engaged with the game for longer and more deeply).
• Attempts by third-party stakeholders to establish independent governance structures face significant legal and economic barriers.
• The long-term sustainability of the esports ecosystem will depend on whether the interests of publishers can be aligned with meaningful cooperation from other stakeholders.

## 5 Conclusion

• The primary aim of this study was to explore the legal and economic structures underpinning the esports industry, with special attention to the recent consolidation period often referred to as the “Esports Winter.” Based on a comparative legal analysis and the interpretation of relevant economic context, the study concludes that this phase is not a collapse, but rather a structural correction that reveals how deeply esports is embedded within the broader video game industry.
• The most important finding is that game publishers retain exclusive control over competition rights, a position grounded in their intellectual property ownership. This legal status creates a fundamental asymmetry that distinguishes esports from traditional sports, where regulatory independence and institutionalized advocacy are standard features. In contrast, popular sports operate within legally unowned domains and enjoy social legitimacy through public institutions. Economically, esports accounts for only a small fraction of total gaming revenues and functions more as a tool for increasing user engagement than as an independent source of profit.
• Efforts to create independent regulatory structures face significant legal and economic constraints. Consequently, the current phase of consolidation should not be interpreted as a sign of decline, but as a transition that more openly and explicitly reflects the publisher-centric model that already defines the ecosystem. Rather than signaling a move toward new frameworks, consolidation stabilizes and renders visible the power dynamics that have always characterized esports. Although some regulatory interventions may improve transparency or player protection, they are unlikely to fundamentally alter the industry's intellectual property-based governance hierarchy.
• Future sustainability does not depend on whether esports can replicate the regulatory models of traditional sports, but on whether collaboration-based frameworks can be developed. These must recognize the central role of publishers while also creating space for other stakeholders to innovate, evolve professionally, and pursue contractual protections. According to the authors, such progress requires atypical agreements between publishers and tournament organizers—agreements that will only materialize if publishers are willing to allow it. In legal terms, publishers remain the “masters of the case.”
